# Construction of Azadirachtin-Loaded Mesoporous Silica Nanoparticles and Their Fast-Acting Insecticidal Activity Against the Mangrove Defoliator *Hyblaea puera*

**DOI:** 10.3390/nano16171083

**Published:** 2026-08-31

**Authors:** Mingcai Wang, Yanxue Liu, Shangkun Gao, Mingshan Chang, Liangming Cao, Yaojun Zhu

**Affiliations:** 1 College of Plant Protection, Shandong Agricultural University, Tai’an 271018, China; 2 Engineering Research Center of Forest Pest Management of Shandong Province, Tai’an 271018, China; 3 Guangxi Forestry Laboratory, Guangxi Zhuang Autonomous Region Forestry Research Institute, Nanning 530002, China; 4 Institute of Forest Ecological Environment and Nature Conservation, Chinese Academy of Forestry Sciences, Haidian, Beijing 100091, China; 5 Guangdong Zhanjiang Mangrove Wetland Ecosystem National Positioning Observation and Research Station, Zhanjiang 524448, China

**Keywords:** mangrove, *Hyblaea puera*, dendritic mesoporous silica nanoparticles, azadirachtin, achieve rapid emergency control

## Abstract

Mangrove ecosystems represent critical coastal blue carbon sinks, whose ecological stability is essential for sustaining carbon sequestration capacity and coastal ecological security. *Hyblaea puera*, a dominant defoliating pest in mangrove habitats, exhibits abrupt population outbreaks and extreme destructive potential, severely threatening the integrity of blue carbon ecosystems. While conventional broad-spectrum chemical insecticides deliver adequate control efficacy, they often exert detrimental effects on non-target organisms and adjacent coastal marine environments, conflicting with core mangrove conservation objectives. Azadirachtin, a botanical insecticide with high lepidopteran target highly selective, is a promising eco-friendly alternative; nevertheless, the dual stresses of periodic tidal flushing and intense ultraviolet radiation in intertidal mangrove habitats drive rapid photodegradation and leaching loss of azadirachtin, leading to poor field persistence and slow lethal action of the free formulation. These limitations render neat azadirachtin insufficient to meet the urgent demand for rapid emergency control of *H. puera* during population outbreaks. To address these technical bottlenecks, we developed an azadirachtin-loaded dendritic mesoporous silica nanoparticle delivery system to simultaneously improve the environmental stability and fast-acting insecticidal efficacy of azadirachtin against *H. puera*. Specifically, dendritic mesoporous silica nanoparticles (DMSNs) were synthesized via a one-pot method, then loaded with azadirachtin (ADT) and further surface-modified with polyether-modified heptamethyltrisiloxane (PM) to fabricate the ADT@DMSN/PM nano-delivery system. The physicochemical properties, intertidal habitat adaptability, insecticidal activity, and environmental safety of the fabricated system were systematically evaluated. Characterization results showed that the as-synthesized DMSNs had a uniform particle size of 200–300 nm, a specific surface area of 260.99 m^2^/g, and an ADT loading efficiency of 66.6%. ADT@DMSNs exhibited prominent alkaline-responsive sustained-release behavior, with a cumulative ADT release of 57.0% at pH 8.2 (mangrove seawater conditions) over 500 h. Laboratory bioassays revealed that the 24 h median lethal concentrations (LC_50_) of ADT@DMSNs against third-, fourth-, and fifth-instar *H. puera* larvae were 84.36, 188.08, and 458.65 μg/mL, respectively, representing 2.38-, 2.63-, and 2.86-fold enhancements in insecticidal activity compared with free ADT. After modification with the PM adjuvant, the nanoformulation exhibited a significantly reduced contact angle on *Avicennia marina* leaves, over 3-fold higher foliar penetration efficiency, and markedly improved tidal wash-off resistance. Field cage bioassays demonstrate that the ADT@DMSN/PM nanoformulation yields a corrected mortality of approximately 95% against *H. puera* larvae at 1200 mg/L at 24 h. This nano-delivery system is precisely tailored to the unique environmental constraints of mangrove intertidal habitats, offering a novel eco-friendly technical approach to achieve rapid emergency control of *H. puera* outbreaks.

## 1. Introduction

Mangrove ecosystems represent a unique blue carbon reservoir in tropical and subtropical intertidal zones, delivering irreplaceable ecological and socioeconomic services such as coastal protection, biodiversity conservation, and climate regulation [1,2]. As a pioneer mangrove species, *A. marina* is instrumental in maintaining the structural stability of intertidal ecosystems; however, its health is severely imperiled by recurrent outbreaks of eruptive defoliating pests [3,4]. Among these, the lepidopteran defoliator *H. puera* has emerged as the primary outbreak species within mangrove reserves in South China. It was first documented in Guangxi mangroves in 2010 and has since undergone recurrent, large-scale eruptions in core protected areas across Guangxi and Hainan. A single female can oviposit up to 1000 eggs, conferring a pronounced capacity for acute, large-area infestations. In Guangxi alone, the mean annual mangrove area affected approaches 500 hm^2^. Sustained defoliation caused by repeated larval feeding severely disrupts the photosynthetic physiology and natural regenerative potential of host plants, thereby directly jeopardizing the stability of the entire mangrove ecosystem [5,6,7,8,9].

The extreme habitat conditions of the mangrove intertidal zone—characterized by periodic tidal inundation, elevated salinity, intense ultraviolet radiation, and high ecological sensitivity have created a unique technical bottleneck for the control of *H. puera*, a challenge without precedent in terrestrial systems. The application of conventional chemical pesticides is strictly prohibited in these habitats for two principal reasons. First, the intertidal zone functions as a critical nexus for material and energy exchange between terrestrial and marine environments; pesticide residues can therefore inflict irreversible damage on non-target aquatic organisms and severely compromise mangrove biodiversity. Second, periodic tidal inundation induces rapid wash-off and dissipation of conventional formulations, which not only precludes effective pest suppression but also exacerbates the risk of offshore pollution [10,11,12]. Therefore, the development of environmentally benign control technologies that are specifically adapted to mangrove intertidal habitats integrating environmental safety, emergency outbreak suppression capacity, and long-term ecological sustainability has become an urgent imperative for the conservation of mangrove ecosystems.

Azadirachtin, a tetranortriterpenoid bioactive compound derived from the neem tree (*Azadirachta indica*), exhibits potent antifeedant, growth-inhibitory, and reproduction-disrupting activities against lepidopteran pests [13,14,15,16]. Its high target highly selective and rapid environmental degradability align closely with the stringent ecological protection requirements of mangrove ecosystems. However, the inherent limitations of azadirachtin namely its poor photothermal stability, weak foliar retention, and short residual activity are substantially amplified under the extreme conditions of mangrove habitats. Intense ultraviolet radiation and elevated temperatures accelerate its photodegradation and thermal inactivation, while the thick epicuticular wax layer of host leaves impedes its cuticular penetration. Moreover, tidal wash-off imposes dual stringent demands on the formulation: ultra-rapid penetration kinetics within the narrow tidal window to enable emergency outbreak suppression, coupled with exceptionally strong foliar adhesion to ensure prolonged efficacy. Conventional azadirachtin preparations are entirely incapable of meeting these multifaceted requirements, there by severely constraining their practical application in mangrove pest management [17,18,19].

Nanocarrier-based delivery technology offers a revolutionary strategy for overcoming the aforementioned bottlenecks. Among the available nanocarrier platforms, dendritic mesoporous silica nanoparticles (DMSNs) represent an ideal candidate for environmentally compatible pesticide delivery, owing to their high loading capacity, favorable biocompatibility, and controllable release properties [20,21,22,23,24]. Compared with liposomes, dendritic mesoporous silica nanoparticles (DMSNs) possess higher physicochemical stability: they are less susceptible to fusion or cargo leakage, and maintain favorable structural stability during long-term storage. In contrast to polymeric nanoparticles, DMSNs feature a more uniform and precisely tunable pore structure, which effectively mitigates the batch-to-batch variability prevalent in polymeric material systems. When benchmarked against metal–organic frameworks (MOFs), DMSNs exhibit superior stability in aqueous environments and more satisfactory biocompatibility. Although MOFs have a high specific surface area, their inherent tendency to degrade under physiological conditions restricts their clinical applications. DMSNs circumvent these drawbacks, integrating the merits of high drug loading capacity, robust structural stability, and excellent biocompatibility [25]. Our group has previously validated the application potential of DMSNs in pesticide delivery systems [26]. However, existing conventional nano-delivery systems have been exclusively developed for terrestrial pest management scenarios and have yet to address the core technical challenges of ultra-rapid penetration and resistance to repeated tidal wash-off in the mangrove intertidal zone. To address this gap, the present study aims to construct a highly permeable, erosion-resistant azadirachtin nano-delivery system adapted to mangrove habitats by fabricating a composite nano-formulation comprising azadirachtin-loaded DMSNs and the permeable polyether-modified heptamethyltrisiloxane (PM) adjuvant (ADT@DMSNs/PM). Owing to its capacity to enhance the permeability of the nano-delivery system, PM facilitates faster penetration of the system into *Avicennia marina* leaves under periodic tidal inundation conditions, thereby increasing the retention efficiency of the nano-system within the leaf tissues [27]. This work is built on the core hypothesis that a rationally engineered nano-delivery system can overcome the three critical bottlenecks limiting azadirachtin efficacy in mangrove environments: rapid environmental inactivation, poor foliar cuticular penetration, and high susceptibility to tidal wash-off. Accordingly, this study aims to experimentally validate whether this delivery strategy enables both efficient emergency suppression and sustainable population management of *Hyblaea puera* in tidal mangrove ecosystems.

## 2. Materials and Methods

### 2.1. Experimental Materials

Tetraethyl orthosilicate (TEOS), triethanolamine (TEA), sodium salicylate, cetyltrimethylammonium bromide (CTAB), and polyether-modified heptamethyltrisiloxane (PM) were purchased from Shanghai Macklin Biochemical Technology Co., Ltd. (Shanghai, China). A 5% azadirachtin (ADT) stock solution was obtained from Ogoni Biotechnology Co., Ltd., and a 97.8% azadirachtin analytical standard was supplied by Tai’an Huiweimin Experimental Instrument Co., Ltd. (Tai’an, China). Fluorescein isothiocyanate (FITC) was acquired from Shanghai Yuanye Biotechnology Co., Ltd. (Shanghai, China). Larvae of the *H. puera*, were field-collected from *A. marina* stands in the Guangxi Beilun Estuary National Nature Reserve and reared to the target instar stages under controlled laboratory conditions.

### 2.2. Experimental Method

#### 2.2.1. Preparation of DMSNs and ADT Loading

Dendritic mesoporous silica nanoparticles (DMSNs) were synthesized via a one-pot method according to the previously established protocol of our group [26]. To prepare the azadirachtin-loaded nanoparticles, 50 mg of DMSN powder was dispersed in 100 mL of methanol, followed by the addition of 10 mL of 5% azadirachtin stock solution. The mixture was magnetically stirred at room temperature for 48 h. Upon completion, the suspension was centrifuged at 8000 rpm for 10 min, the supernatant was discarded, and the resulting precipitate was washed three times with methanol and subsequently freeze-dried to obtain ADT-loaded DMSNs (ADT@DMSNs). The morphology, particle size, ζ-potential, crystalline structure, pore architecture, and thermal stability of DMSNs and ADT@DMSNs were characterized using scanning electron microscopy (SEM, SM-7800, Hitachi High-Tech Corporation, Tokyo, Japan), transmission electron microscopy (TEM, HT7800, Hitachi High-Tech Corporation, Tokyo, Japan), dynamic light scattering (DLS, Nano Brook90Plus PALS, Brookhaven Instruments Corporation, Nashua, NH, USA), X-ray diffraction (XRD, Rigaku Corporation, Tokyo, Japan), nitrogen adsorption–desorption analysis (TriStar II Plus 3030, Micromeritics Instrument Corporation, Norcross, GA, USA), and thermogravimetric analysis (TGA, STA7300, Hitachi High-Tech Analysis Corporation, Shizuoka, Japan).

#### 2.2.2. Determination of Drug Loading Rate of ADT@DMSNs

The drug loading content of ADT@DMSNs was determined by ultraviolet (UV) spectrophotometry. A standard stock solution of azadirachtin (160 μg/mL) was prepared by dissolving 4 mg of the 97.8% azadirachtin standard in methanol and diluting to a final volume of 25 mL. A series of working standard solutions at concentrations of 8, 16, 20, 40, and 80 μg/mL were obtained by gradient dilution. The absorbance of each solution was measured at 234 nm, and a calibration curve was constructed. The supernatant collected during the drug loading process was appropriately diluted with methanol, and its absorbance was measured at 234 nm. The concentration of free ADT in the supernatant was determined from the calibration curve. The drug loading content was calculated using the following formula:

(1) $$\mathrm{Drug}\;\mathrm{loading}\;\mathrm{rate}\;(\%)\;=\;(\frac{m_{1}-m_{2}}{m_{0}\;+{\;m}_{1}\;-\;m_{2}})\;\times \;100\%$$

where m_0_ is the total mass of ADT and DMSNs, m_1_ is the total mass of ADT, m_2_ is the mass of free ADT, (m_1_ − m_2_) represents the mass of ADT loaded into DMSNs, and (m_0_ + m_1_ − m_2_) corresponds to the total mass of ADT@DMSNs.

#### 2.2.3. Analysis of Sustained Release Performance of ADT@DMSNs

In vitro release studies were performed by uniformly dispersing 20 mg of ADT@DMSN powder in 50 mL of phosphate-buffered saline (PBS) at pH 6.0, 7.4, and 8.2, respectively, to simulate the pH conditions of the mangrove habitat and the insect internal environment. The dispersions were incubated in a thermostatic orbital shaker maintained at 27 °C with continuous agitation at 150 rpm. At predetermined time intervals (3, 6, 12, 24, 72, 120, 168, 336, and 500 h), samples were withdrawn and centrifuged at 8000 rpm for 10 min. The pellet was collected, washed with methanol, and centrifuged again. The combined supernatant fractions were used to measure absorbance at 234 nm. The cumulative amount of ADT released was calculated from the standard curve, and the cumulative release profile was plotted.

#### 2.2.4. Indoor Toxicity Determination of ADT@DMSNs

The insecticidal activity against third-, fourth-, and fifth-instar larvae of *H. puera*, selected for uniform size and vigorous health, was evaluated. Based on preliminary experimental results, five gradient concentrations of ADT@DMSN suspension and free ADT solution were established, with distilled water serving as the control. An aliquot of 2 mL of each treatment solution was uniformly applied to a filter paper placed in a Petri dish. After the filter paper was completely saturated, eight larvae were introduced into each dish, with three replicates per treatment. The larvae were maintained at 27 °C, 75% relative humidity, and a photoperiod of 14L:10D. Larval mortality was recorded at 1, 3, 6, 12, 24, 36, and 48 h post-treatment, and corrected mortality was calculated. Probit analysis was performed using SPSS 26.0 software to derive the toxicity regression equations and the 24 h median lethal concentration (LC_50_) values.

#### 2.2.5. Fluorescence Labeling and Biodistribution Determination

Fluorescent probes were prepared by labeling DMSNs with fluorescein isothiocyanate (FITC). Briefly, 10 mg of FITC was dispersed in 100 mL of ultrapure water, followed by the addition of 100 mg of DMSNs powder. The mixture was stirred at room temperature in the dark for 24 h. The resulting precipitate was collected by centrifugation, washed five times with ultrapure water to remove unbound FITC, and subsequently freeze-dried to obtain FITC@DMSNs.

To track the tissue distribution, a suspension of FITC@DMSNs/PM was prepared by blending fluorescein isothiocyanate-labeled DMSNs (FITC@DMSNs) with the polyether-modified heptamethyltrisiloxane (PM) adjuvant at the specified ratio. Healthy fifth-instar larvae of *H. puera* were fully immersed in the FITC@DMSNs/PM suspension. After 3 h of exposure, the larvae were dissected, and the midgut tissues were fixed in 4% paraformaldehyde and processed into paraffin sections. In parallel, fresh leaves of *A. marina* were treated with FITC@DMSN and FITC@DMSN/PM suspensions; after 3 h, leaf cross-sections were obtained and similarly embedded in paraffin. The spatial distribution of DMSNs within the larval midgut and leaf tissues was subsequently examined using fluorescence microscopy.

#### 2.2.6. Evaluation of Foliar Wettability and Permeability

The static contact angles of different test liquids on the leaf surface of *A. marina* were measured using a contact angle goniometer. Fresh leaves were affixed onto glass slides, and 5 μL droplets of ultrapure water, ADT@DMSN suspension, and ADT@DMSN/PM suspension were deposited individually. The contact angle values were derived by the circular curve fitting method.

The permeation and diffusion behavior of DMSNs on the leaf surface was visualized using in vivo fluorescence imaging. Suspensions of FITC@DMSNs and FITC@DMSNs/PM were applied to the adaxial surface of *A. marina* leaves, and fluorescence images were captured with a small animal imaging system at 0 s, 30 s, 1 min, and 5 min to analyze the time-dependent permeation and diffusion characteristics.

#### 2.2.7. Evaluation of Resistance to Tidal Wash-Off

The resistance of the formulations to tidal wash-off was evaluated through a simulated tidal immersion assay. Aliquots of 50 μL of ADT@DMSN suspension and 10, 20, and 50 μL of ADT@DMSN/PM suspension were separately deposited onto the surface of fresh *A. marina* leaves. After air-drying at room temperature, the surface morphology was imaged using a digital microscope with ultra-depth of field at magnifications of ×200 and ×500. Subsequently, the leaves were immersed in artificial seawater for 30 min to simulate a single tidal inundation event. Upon removal and air-drying, the leaf surfaces were re-imaged to compare the residual distribution of nanoparticles before and after immersion.

#### 2.2.8. Forest Field Trial

This study was performed in a natural stand of *A. marina*, where healthy branches with consistent growth vigor were selected as experimental units. Three treatments were set, including blank control CK, (deionized water), ADT, and ADT@DMSNs/PM, each prepared at two concentration gradients (600 mg/L and 1200 mg/L). Bag-shaped test cages fabricated from 80-mesh nylon insect netting were secured onto the selected branches. Four biological replicates were assigned to each treatment, with 30 *H. puera* larvae randomly placed into each cage per replicate. Uniform foliar spraying was applied across all leaf surfaces for each treatment. After the spray solution had air-dried naturally on the foliage, the test larvae were introduced into the cages, which were then sealed and fastened securely. All cages were maintained in the in-situ mangrove habitat throughout the experiment and subjected to periodic inundation by natural tides. At 24 h post-treatment, the number of dead larvae in each cage was recorded, and the corrected mortality was calculated for each treatment.

#### 2.2.9. Statistical Analysis and Drawing

IBM SPSS statistical software was used to analyze the experimental data, and the larval mortality rate, corrected mortality rate, virulence regression equation and LC_50_ value were calculated. Drawing with Origin 2021.

## 3. Results and Discussion

### 3.1. Structural Characterization and Physicochemical Properties of DMSNs and ADT@DMSNs

**Figure 1:**
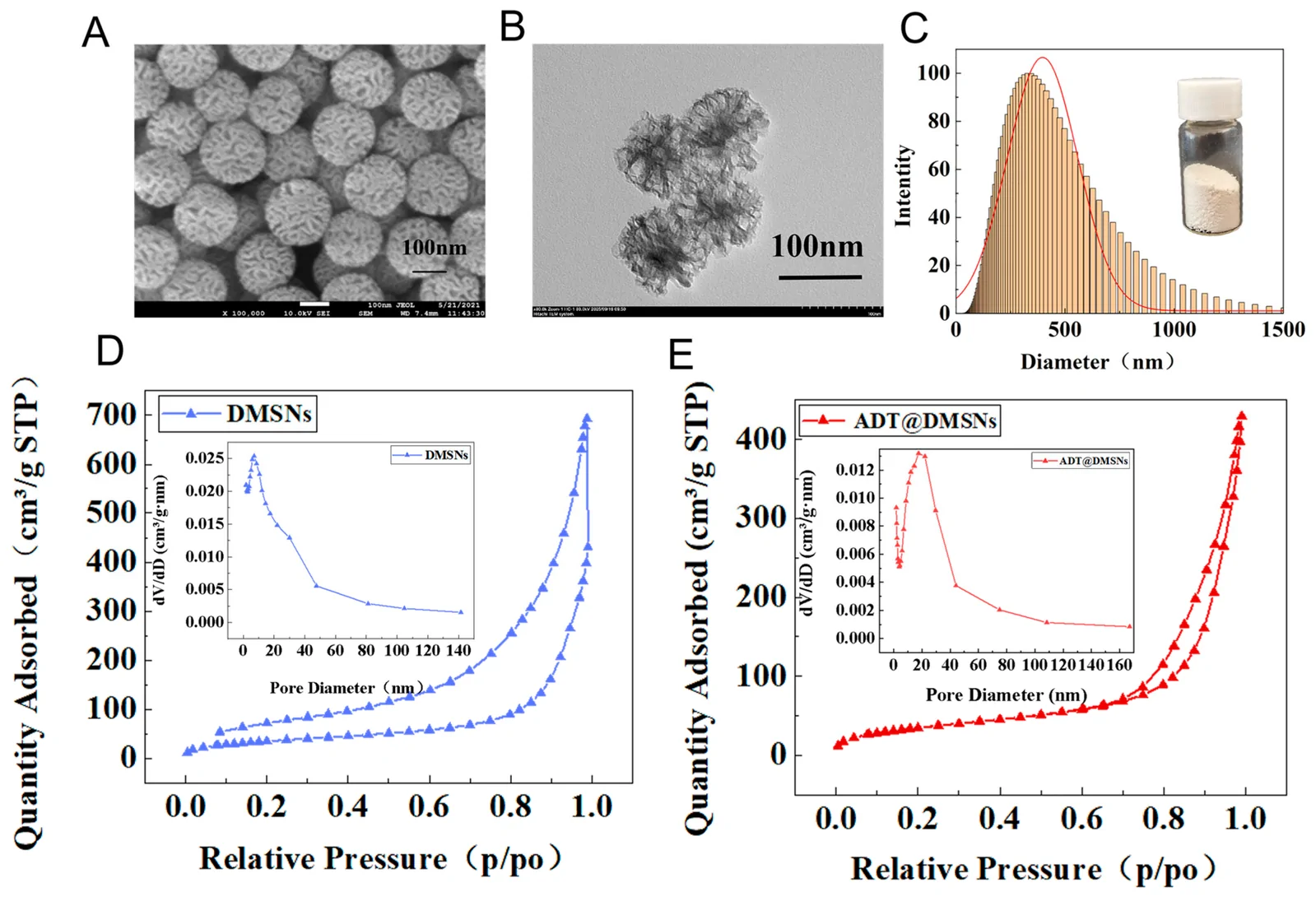
(**A**) DMSN scanning electron microscopy; (**B**) Transmission electron microscopy of DMSNs; (**C**) DMSN particle size distribution; (**D**) the nitrogen adsorption/desorption curves and pore size distribution of DMSNs; (**E**) the nitrogen adsorption/desorption curves and pore size distribution of ADT@DMSNs.

Characterization by TEM and complementary techniques revealed that the as-synthesized DMSNs possessed a highly uniform size distribution, with particle diameters ranging from 200 to 300 nm (Figure 1A–C). The particles exhibited a characteristic dendritic morphology featuring radially oriented, wrinkled pore channels that penetrated directly into the particle interior; the pore walls were relatively thin, and the overall pore architecture was highly accessible. DLS measurements yielded a ζ-potential of −38.66 mV, the high absolute value of which indicates excellent dispersion stability in aqueous media and a pronounced resistance to aggregation.

Nitrogen adsorption–desorption measurements revealed that the DMSNs displayed a characteristic Type IV isotherm (IUPAC classification) with a pronounced hysteresis loop in the medium-to-high relative pressure region (P/P_0_ = 0.4–0.9) (Figure 1D), confirming the presence of a well-developed mesoporous framework. The adsorption capacity continued to increase as P/P_0_ approached 1.0, indicating the existence of a certain amount of micropores in addition to the mesopores, which is consistent with the hierarchical dendritic pore architecture observed by TEM. The nitrogen adsorption–desorption isotherm of ADT@DMSNs retained a similar profile and still conformed to the IUPAC Type IV classification (Figure 1E), demonstrating that the mesoporous skeleton remained intact after drug loading. The average pore diameter of DMSNs was determined to be 17.10 nm, with a specific surface area of 260.99 m^2^/g. Following ADT loading, the average pore diameter of ADT@DMSNs decreased to 12.68 nm, and the specific surface area was significantly reduced to 129.90 m^2^/g. The concurrent decline in both specific surface area and pore size provides robust evidence that ADT molecules were successfully accommodated within the internal mesoporous channels of DMSNs, achieving efficient loading.

**Figure 2:**
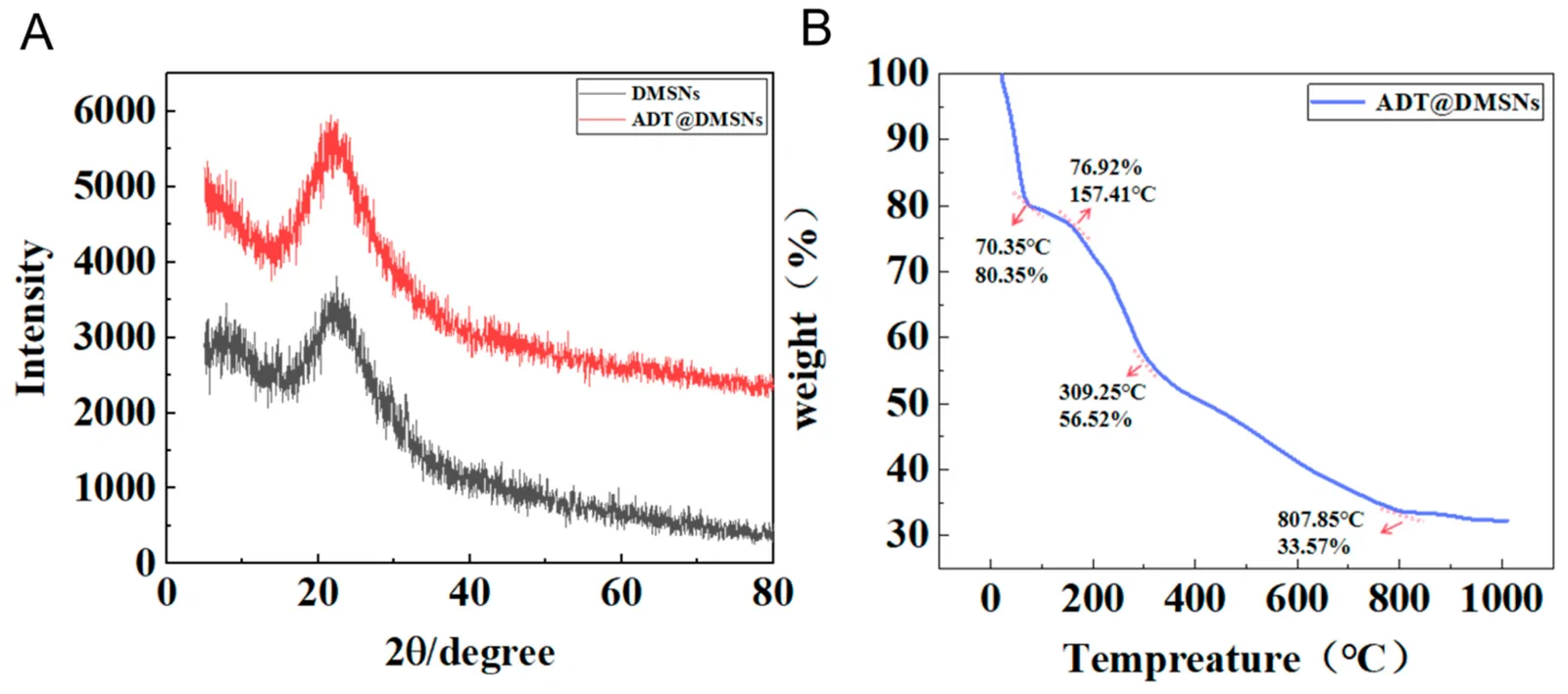
(**A**) X-ray diffraction (XRD) patterns of DMSNs and ADT@DMSNs; (**B**) Thermogravimetric analysis (TGA) curve of ADT@DMSNs.

The XRD pattern of DMSNs exhibited a broad, diffuse diffraction peak centered at 2θ ≈ 22°, with no other sharp characteristic reflections (Figure 2A). This feature is in complete agreement with the standard XRD signature of amorphous silica, confirming that the as-synthesized DMSNs possess an amorphous silica structure. A comparison with the XRD pattern of ADT@DMSNs revealed no appreciable changes in peak position, profile, or intensity, demonstrating that the physical loading of ADT did not compromise the bulk amorphous framework of the DMSNs.

The TGA profile of ADT@DMSNs (Figure 2B) exhibited a multi-stage thermal decomposition behavior. An initial weight loss of 19.65% was recorded up to 70.35 °C, predominantly ascribed to the removal of surface-adsorbed water and intrapore bound water. At 157.41 °C, the residual mass was 76.92%, with the weight loss in this stage principally attributed to the thermal decomposition of ADT molecules. The sample retained more than 50% of its initial mass at 300 °C, and the mass remained essentially constant upon further heating to 800 °C. These findings collectively demonstrate that ADT@DMSNs possesses excellent thermal stability, thereby meeting the practical application requirements under the high-temperature conditions characteristic of mangrove habitats.

### 3.2. Analysis of Drug Loading and Release Properties of ADT@DMSNs

A standard calibration curve was constructed using azadirachtin-methanol standard solutions, yielding the linear regression equation (y = 0.0122x + 0.0595) with a correlation coefficient (R^2^ = 0.9972). This high linearity confirms an excellent linear relationship between ADT concentration and absorbance over the range of 8–160 μg/mL, thereby ensuring accurate quantification of drug loading content and subsequent release profiles. Based on this calibration curve, the drug loading content of ADT onto DMSNs was determined to be as high as 66.6%, a value substantially exceeding that typically achieved with conventional mesoporous silica carriers. This superior loading performance is primarily attributed to the unique dendritic, radially oriented pore architecture and the large specific surface area of DMSNs, which provide abundant adsorption sites for ADT molecules, while the highly open pore channels facilitate efficient molecular diffusion and adsorption.

**Figure 3:**
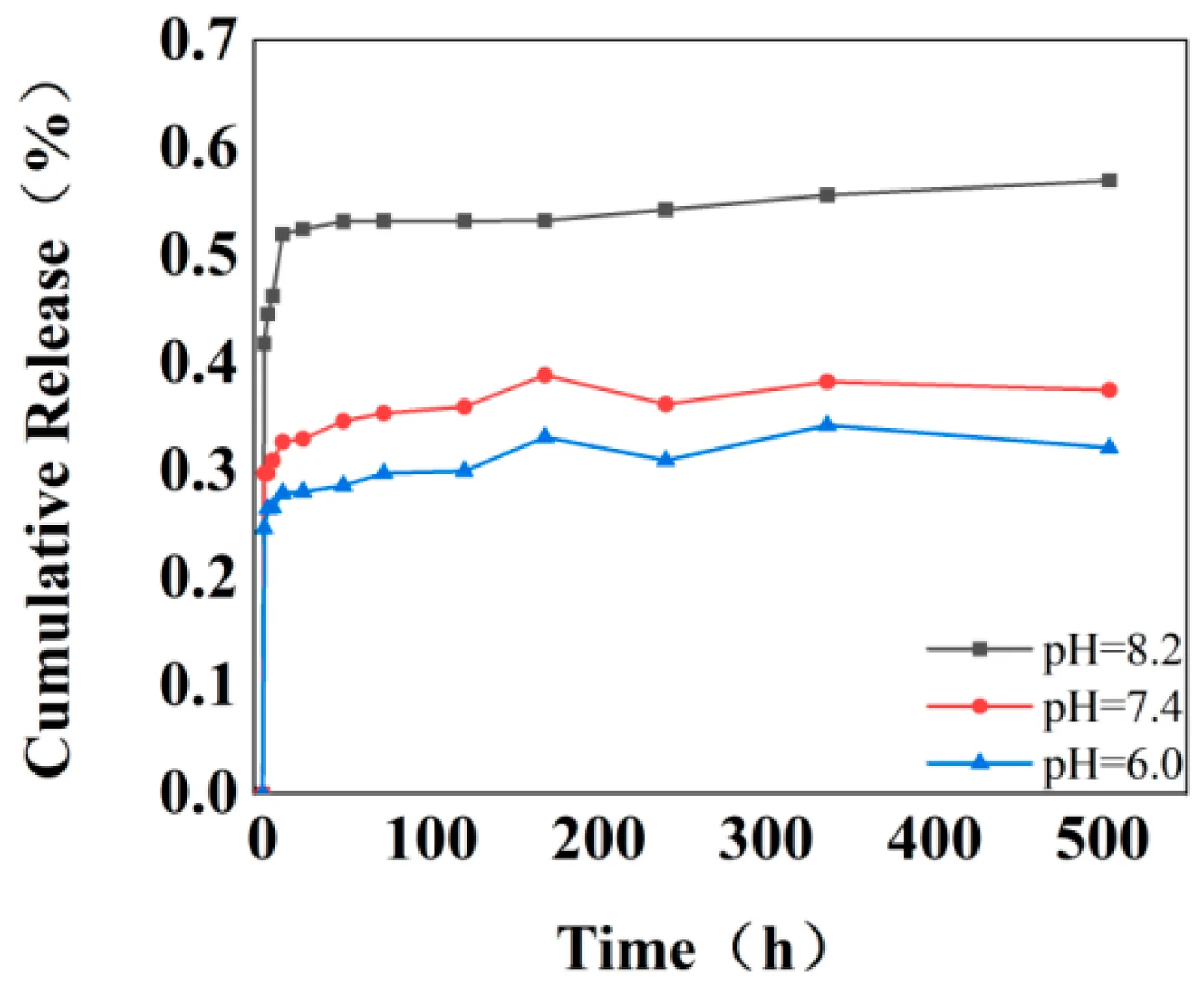
The sustained release diagram of ADT@DMSNs at 25 °C and different pH conditions. Note: pH 6.0, 7.4, and 8.2 simulate acidic soil, insect hemolymph, and mangrove seawater environments, respectively.

The in vitro cumulative release profiles of ADT@DMSNs under different pH conditions are presented in Figure 3 The results revealed a pronounced pH-dependent release behavior: the fastest release was observed at pH 8.2, with a cumulative release of 56.96% at 168 h and approximately 57.0% at 500 h; a moderate release rate was recorded at pH 7.4, reaching approximately 37.5% cumulative release at 500 h; and the slowest release occurred at pH 6.0, yielding only 32.0% cumulative release at 500 h. This alkaline-sensitive release characteristic is primarily attributed to the gradual hydrolytic degradation of the amorphous silica framework under alkaline conditions, which progressively disrupts the mesoporous architecture and accelerates the liberation of encapsulated ADT. Given that the pH of mangrove seawater typically ranges from 8.0 to 8.5, the alkaline-responsive release profile of ADT@DMSNs is expected to enable sustained release of the active ingredient within the mangrove habitat, thereby prolonging the residual efficacy. Concurrently, the weakly alkaline environment of the insect midgut is also conducive to the release of ADT, potentially enhancing insecticidal activity.

### 3.3. Indoor Toxicity Determination of ADT@DMSNs

**Figure 4:**
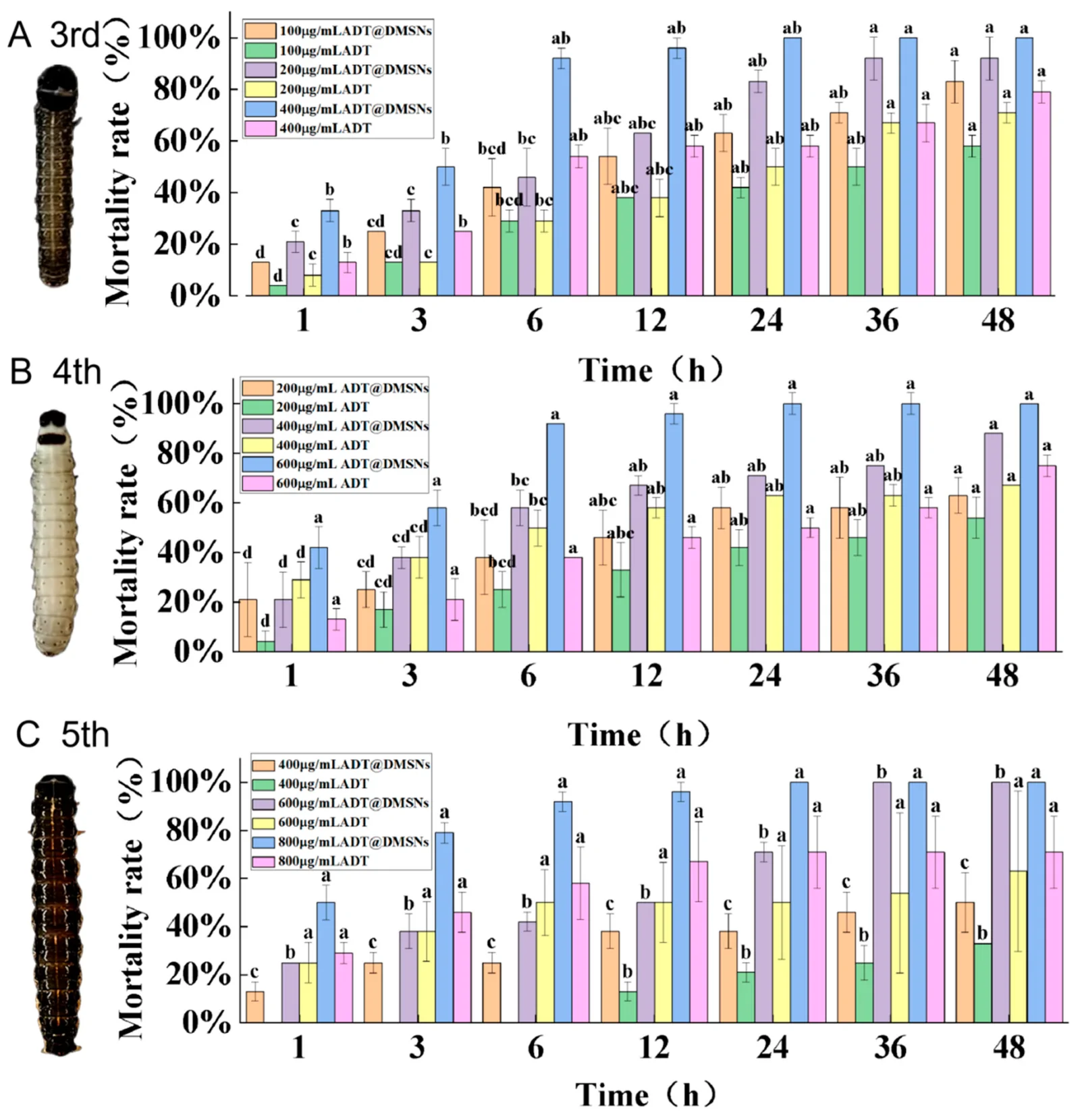
Time-dose toxicity profiles of ADT@DMSNs and free ADT against *H. puera* larvae. (**A**) Third-instar larvae; (**B**) Fourth-instar larvae; (**C**) Fifth-instar larvae. Note: Data are presented as mean ± standard error (*n* = 3). Different lowercase letters indicate significant differences among treatments at the same time point (*p* < 0.05, Duncan’s multiple range test).

At equivalent concentrations and time points, the contact toxicity mortality of ADT@DMSNs was consistently and significantly higher than that of free ADT, and this enhancement was particularly pronounced at lower concentrations and during the early phase of exposure (1–12 h). Specifically, Figure 4A third-instar larvae treated with 400 μg/mL ADT@DMSNs reached 90.0% mortality at 6 h, compared to only 55.0% for free ADT at the same concentration; Figure 4B fourth-instar larvae exposed to 600 μg/mL ADT@DMSNs attained 95.0% mortality at 12 h, markedly exceeding the 48.0% recorded in the free ADT group; Figure 4C fifth-instar larvae subjected to 800 μg/mL ADT@DMSNs achieved 98.0% mortality at 24 h, whereas free ADT gave merely 70.0%. This nanocarrier-mediated potentiation of insecticidal activity has been widely corroborated in lepidopteran pest control, and its central mechanism resides in the ability of mesoporous silica to breach the insect cuticular barrier through physical penetration and transmembrane transport, thereby substantially improving the bioavailability of the active ingredient [28,29,30,31].

Probit analysis revealed that the 24 h median lethal concentrations (LC_50_) of ADT@DMSNs against third-, fourth-, and fifth-instar larvae of *H. puera* were 84.36, 188.08, and 458.65 μg/mL (95% confidence intervals: 72.15–96.57, 165.32–210.84, and 421.56–495.74 μg/mL), representing 2.38-, 2.63-, and 2.86-fold greater toxicity than that of free ADT, respectively. Larval susceptibility decreased markedly with increasing instar, a phenomenon closely associated with the thicker cuticular structure and elevated detoxification enzyme activity in older larvae, suggesting that field applications should preferentially target early instars (≤3rd instar) to achieve optimal control efficacy. Furthermore, ADT@DMSNs exhibited both superior rapid action and prolonged residual activity relative to free ADT, attaining 50% mortality 6–12 h earlier and maintaining significantly higher efficacy at 24–48 h post-treatment, without the rapid efficacy decline typically encountered with conventional azadirachtin formulations. This performance corroborates the encapsulation and controlled-release function conferred by the dendritic mesoporous architecture of DMSNs.

### 3.4. Foliar Penetration and In Vivo Insect Delivery Performance of DMSNs

**Figure 5:**
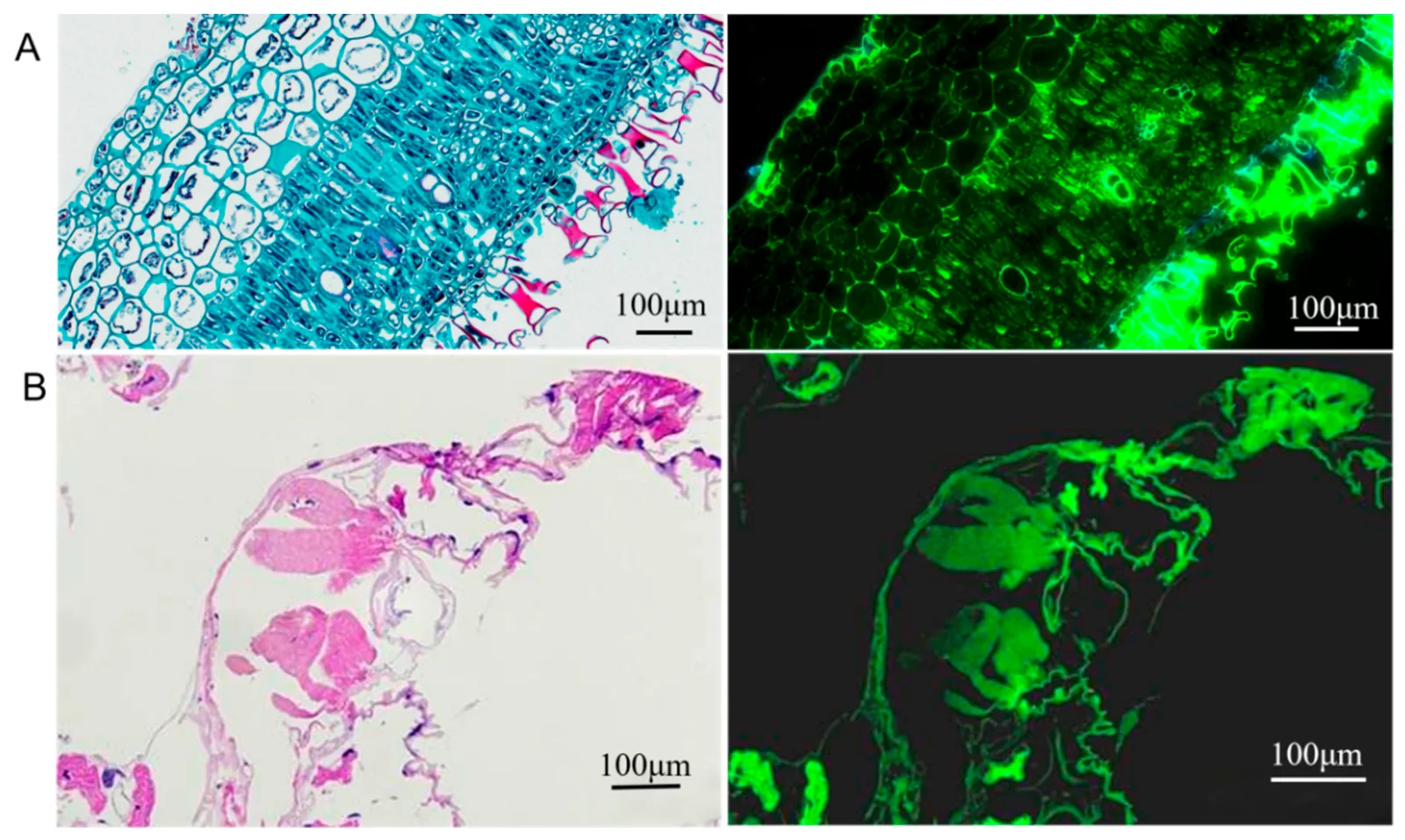
Biodistribution of fluorescently labeled DMSNs in *A. marina* leaves and the larval midgut of *H. puera*. (**A**) Cross-section of *A. marina* leaf (left: bright-field image; right: fluorescence image). (**B**) Histological section of the midgut of a fifth-instar *H. puera* larva (left: hematoxylin and eosin (HE) staining; right: fluorescence image). Note: Scale bar = 100 μm; green fluorescence signals indicate FITC-labeled DMSNs.

To evaluate the delivery potential of DMSNs within the mangrove ecosystem, *A. marina* leaves and fifth-instar larvae of *H. puera* were treated with FITC-labeled DMSNs, and their biodistribution was subsequently examined by fluorescence microscopy (Figure 5). As shown in Figure 5A, the bright-field image clearly resolved the anatomical structures of the *A. marina* leaf, including the cuticular wax layer, epidermis, and spongy mesophyll. In the corresponding fluorescence image, intense green fluorescence signals were uniformly distributed throughout the spongy mesophyll tissue. These observations indicate that DMSNs can effectively penetrate the thick cuticular wax layer and epidermis of *A. marina* leaves and reach the underlying photosynthetic tissues.

Figure 5B presents the histological sections of the larval midgut of *H. puera*. The hematoxylin and eosin (HE)-stained image reveals the typical morphological architecture of the larval midgut, while the corresponding fluorescence image shows distinct green fluorescent signals distributed within the midgut tissue. This observation confirms that DMSNs can penetrate the insect integument and successfully reach the midgut—the primary target organ of azadirachtin—thereby further elucidating the mechanistic basis for the enhanced insecticidal activity of ADT@DMSNs. The superior penetration capability of DMSNs across both plant leaf tissues and the insect cuticle lays a solid foundation for their application as nanocarriers in mangrove pest management. By substantially improving the delivery efficiency of active ingredients such as azadirachtin, DMSNs can enable a significant reduction in pesticide application rates, thereby minimizing adverse impacts on non-target organisms and the surrounding environment.

### 3.5. Effect of PM Adjuvant on Foliar Wettability and Penetration Efficiency of DMSNs

**Figure 6:**
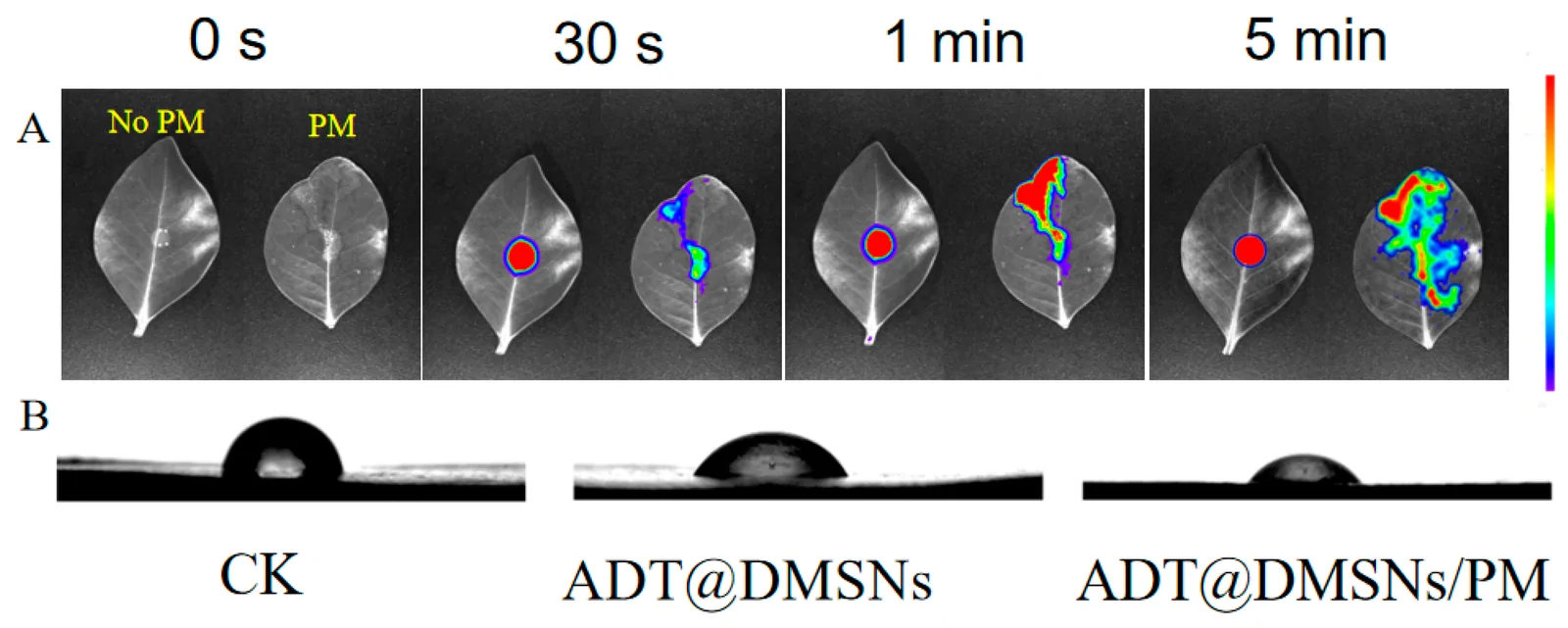
Effect of PM Adjuvant on the Foliar Wettability and Penetration Performance of DMSNs on *A. marina* Leaves. (**A**) In vivo fluorescence imaging showing the time-dependent permeation and diffusion behavior of FITC@DMSNs (left column) and FITC@DMSNs/PM (right column) at 0 s, 30 s, 1 min, and 5 min. (**B**) Static contact angles of pure water (CK), ADT@DMSNs, and ADT@DMSNs/PM on the leaf surface of *A. marina*. Note: Data are presented as mean ± standard error (*n* = 5); PM, polyether-modified heptamethyltrisiloxane; FITC, fluorescein isothiocyanate. The color gradient from blue to red on the pseudocolor scale corresponds to a continuous increase in fluorescence signal intensity.

To further enhance the delivery efficiency of DMSNs on mangrove leaf surfaces, the effect of the PM adjuvant on the wettability and penetration behavior of DMSNs was investigated (Figure 6A). In vivo fluorescence imaging revealed that at 0 s, the fluorescent signals in both groups were strictly confined to the droplet deposition site. Over time, the fluorescence gradually spread from the deposition point along the leaf venation to other regions of the leaf. Notably, at all time points, the fluorescence diffusion rate and extent in the FITC@DMSN/PM group were substantially greater than those in the FITC@DMSN group. By 5 min, the fluorescent signal in the PM-supplemented group had spread across nearly half of the leaf, whereas in the control group it remained largely restricted to the vicinity of the application site. These observations indicate that the addition of the PM adjuvant markedly improves the penetration and translocation efficiency of DMSNs on mangrove leaves.

The static contact angle measurements (Figure 6B) revealed that the pure water control exhibited the largest contact angle (112.3°), confirming the highly hydrophobic nature of the *A. marina* leaf surface, a common adaptation of mangrove plants to saline environments. The ADT@DMSN formulation showed a slightly reduced contact angle (98.7°) relative to pure water, whereas the ADT@DMSN/PM formulation achieved a dramatically decreased contact angle of 32.5°, indicating optimal wettability. PM is a highly effective organosilicon surfactant capable of substantially lowering the surface tension of aqueous solutions, thereby improving wettability and spreading on hydrophobic leaf surfaces [32,33,34]. In mangrove habitats, where the tidal window typically lasts only 2–4 h, the ultra-fast penetration facilitated by the PM adjuvant ensures that the active agent can fully infiltrate the leaf interior before the onset of tidal inundation, effectively preventing wash-off by seawater.

### 3.6. Effect of PM Adjuvant on the Tidal Wash-Off Resistance of ADT@DMSNs

**Figure 7:**
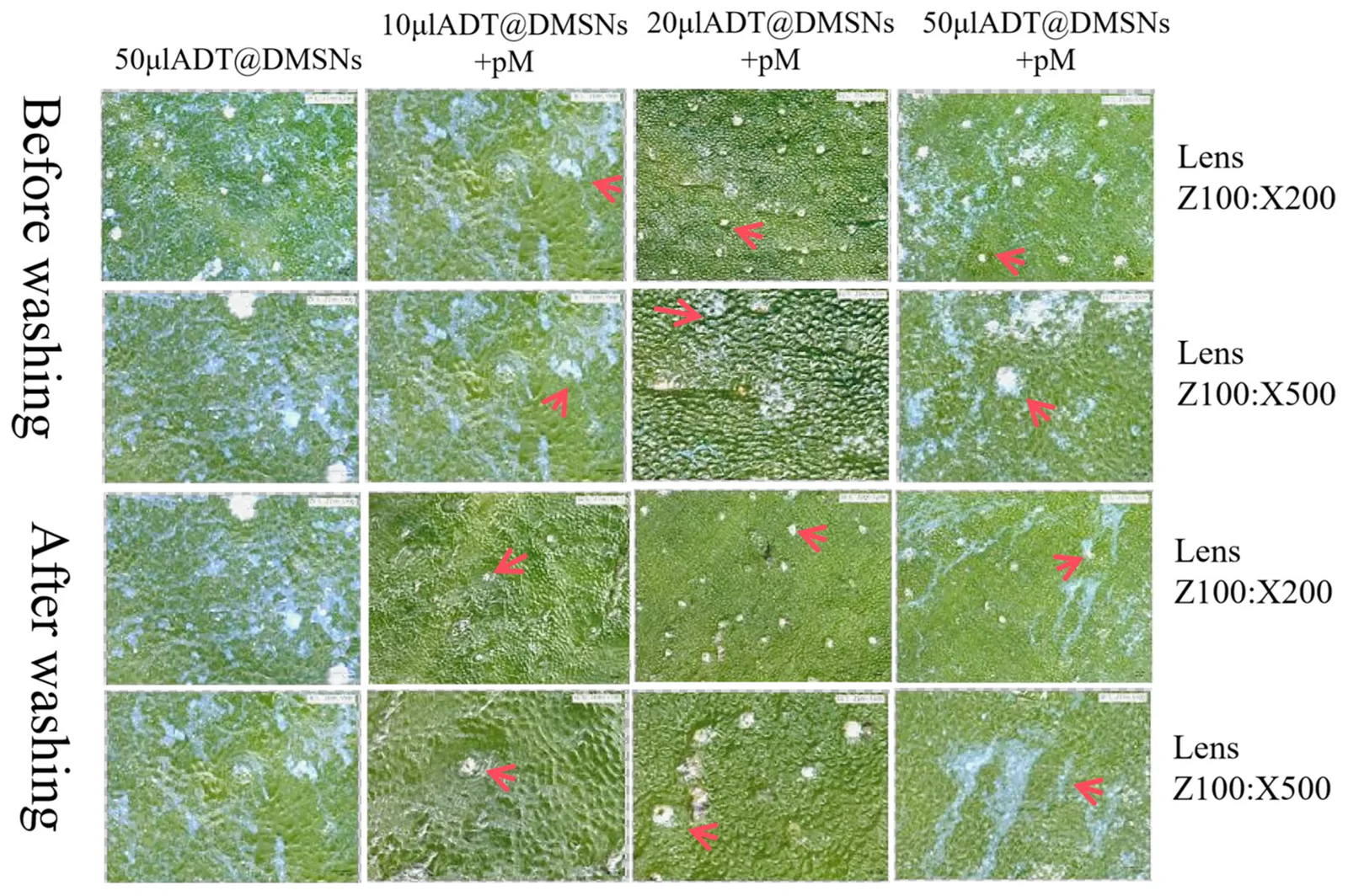
Residual deposition of ADT@DMSNs on the leaf surface of *A. marina* before and after simulated tidal seawater immersion. Columns from left to right: 50 μL ADT@DMSNs (without PM), 10 μL ADT@DMSNs/PM, 20 μL ADT@DMSNs/PM, 50 μL ADT@DMSNs/PM. Rows from top to bottom: before immersion ×200, before immersion ×500, after immersion ×200, after immersion ×500. Note: Red arrows indicate the deposition sites of ADT@DMSNs; blue regions represent nanoparticles that have penetrated into the leaf interior.

Tidal seawater inundation is the primary driver of pesticide wash-off in mangrove ecosystems. The wash-off resistance of ADT@DMSNs formulations with and without the PM adjuvant was evaluated through a simulated tidal immersion assay (Figure 7). Prior to simulated tidal immersion, the 50 μL ADT@DMSN group (without PM) exhibited only a few scattered white deposition spots on the leaf surface of *A. marina*, with virtually no discernible blue penetration zones, indicating poor penetration efficiency even at a high application volume. In contrast, the ADT@DMSN/PM group demonstrated markedly enhanced penetration performance; as the application volume increased from 10 μL to 50 μL, the blue penetration zones on the leaf surface progressively expanded and the deposition sites became more distinct.

Following simulated tidal seawater immersion, virtually all deposition sites on leaves treated with ADT@DMSNs (without PM) had disappeared, and no residual penetration zones were observed, indicating extremely poor tidal wash-off resistance. This is attributable to the fact that the majority of ADT@DMSNs were merely physically adsorbed onto the hydrophobic leaf surface without penetrating the cuticle, rendering them highly susceptible to removal by flowing seawater during tidal inundation. In stark contrast, all ADT@DMSN/PM groups retained conspicuous blue penetration zones and well-defined deposition sites after tidal immersion, with residue levels increasing proportionally with application volume. The 20 μL and 50 μL treatments exhibited the most pronounced retention efficacy.

This marked improvement in tidal wash-off resistance is attributed to the ability of the PM adjuvant to promote the penetration of ADT@DMSNs into the leaf cuticle and epidermis, where the nanoparticles are firmly entrapped and shielded from seawater scouring during tidal cycles. These results demonstrate that the PM adjuvant not only enhances the penetration efficiency of ADT@DMSNs but also provides a fundamental solution to the issue of pesticide loss caused by tidal wash-off, substantially improving pesticide utilization efficiency while mitigating the risk of contamination to marine ecosystems.

### 3.7. Forest Field Trial

**Figure 8:**
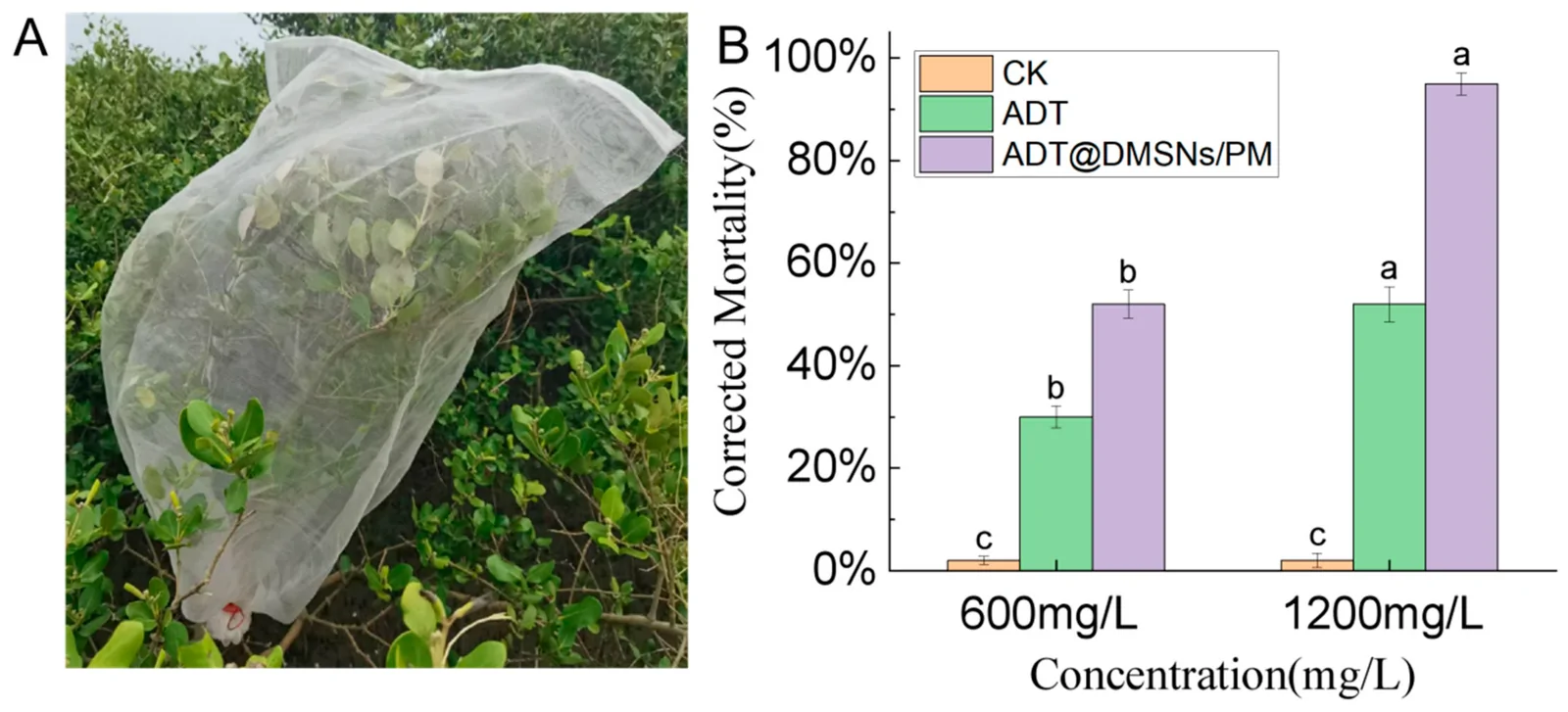
Cage experimental setup in mangrove stands and corrected mortality of *H. puera* larvae under different treatments within 24 h. Figure (**A**) shows the cage bioassay device fixed on branches of *A. marina*. This device preserves the natural field microhabitat while preventing larval escape, natural enemy invasion, and interference from external pesticide Fdrift. Figure (**B**) presents the corrected mortality of *H. puera* larvae at two concentration gradients (600 mg/L and 1200 mg/L). Treatments include CK, ADT, and ADT@DMSNs/PM. Note: Error bars represent standard error (*n* = 4). Different lowercase letters above bars at the same concentration indicate significant differences among treatments (*p* < 0.05, Duncan’s multiple range test).

The corrected mortality in the CK remained consistently below 5%, which met the quality control criteria for pesticide field efficacy trials and verified the stability and reliability of the experimental system. ADT exhibited a significant dose-dependent insecticidal activity, with corrected mortality rates of approximately 30% and 51% at concentrations of 600 mg/L and 1200 mg/L, respectively (Figure 8). In comparison, the ADT@DMSN/PM nanoformulation achieved significantly higher corrected mortality than free azadirachtin at the same concentration level (*p* < 0.05). Specifically, at 1200 mg/L, the corrected mortality of the ADT@DMSN/PM treatment reached approximately 95%, and its control efficacy was nearly doubled relative to that of free azadirachtin at the same concentration. These findings confirm that after tidal inundation, the ADT@DMSN/PM nano-delivery system can significantly enhance the field insecticidal activity of azadirachtin. The underlying synergistic mechanism is mainly attributed to the system improves the foliar adhesion of the active ingredient to resist seawater scouring, and it promotes the penetration of the active ingredient through the insect cuticle. This nanoformulation shows favorable application prospects in the green management of *H. puera*.

## 4. Conclusions

In this study, an azadirachtin nano-delivery system, ADT@DMSNs/PM, tailored to the mangrove intertidal habitat was successfully constructed, effectively resolving the core bottlenecks encountered by conventional azadirachtin formulations in mangrove environments. The main conclusions are as follows:

1. The DMSNs synthesized via a one-pot method exhibited a uniform particle size (200–300 nm), a large specific surface area (260.99 m^2^/g), and a unique dendritic pore architecture, achieving a high ADT loading capacity of 66.6%, together with excellent thermal stability and alkaline-sensitive sustained-release characteristics.
2. ADT@DMSNs exhibited significantly higher toxicity against *H. puera* larvae compared with free ADT, with 24 h LC_50_ values of 84.36, 188.08, and 458.65 μg/mL for third-, fourth-, and fifth-instar larvae, representing 2.38-, 2.63-, and 2.86-fold greater toxicity, respectively.
3. FITC-labeled DMSNs can effectively penetrate the thick cuticular wax layer and epidermis of *A. marina* leaves to reach the spongy mesophyll tissue, and can also traverse the integument of fifth-instar *H. puera* larvae to reach the midgut tissue—the primary target organ of azadirachtin. DMSNs exhibit superior penetration capacity across both plant leaf tissues and insect integuments.
4. After incorporation of the PM adjuvant, the ADT@DMSN formulation demonstrated markedly enhanced foliar wettability and penetration efficiency on *A. marina* leaves, together with substantially improved resistance to tidal wash-off, thereby enabling retention of the majority of the active ingredient following tidal immersion.
5. These findings confirm that after tidal inundation, the ADT@DMSN/PM nano-delivery system can significantly enhance the field insecticidal activity of azadirachtin. The underlying synergistic mechanism is mainly attributed to the system improves the foliar adhesion of the active ingredient to resist seawater scouring, and it promotes the penetration of the active ingredient through the insect cuticle. This nanoformulation shows favorable application prospects in the green management of *H. puera*.

This nano-delivery system is precisely tailored to the specific exigencies of mangrove intertidal habitats, providing a novel technological strategy for the environmentally sustainable management of the teak defoliator, *H. puera*, and offering a valuable reference for the design and development of nanopesticides under extreme habitat conditions.

## Data Availability

Data available in a publicly accessible repository.
